# Medication adherence and tolerability of Alzheimer’s disease medications: study protocol for a randomized controlled trial

**DOI:** 10.1186/1745-6215-14-125

**Published:** 2013-05-04

**Authors:** Noll L Campbell, Paul Dexter, Anthony J Perkins, Sujuan Gao, Lang Li, Todd C Skaar, Amie Frame, Hugh C Hendrie, Chris M Callahan, Malaz A Boustani

**Affiliations:** 1 Department of Pharmacy Practice, Purdue University School of Pharmacy, 410 West 10th Street, West Lafayette, IN, USA; 2 Indiana University Center for Aging Research, Indianapolis, IN, USA; 3 Regenstrief Institute, Inc., Indianapolis, IN, USA; 4 Wishard Health Services, Indianapolis, IN, USA; 5 Department of Medicine, Indiana University School of Medicine, Indianapolis, IN, USA; 6 Department of Medical and Molecular Genetics, Indiana University School of Medicine, Indianapolis, IN, USA; 7 Division of Clinical Pharmacology, Department of Medicine, Indiana University School of Medicine, Indianapolis, IN, USA; 8 Department of Psychiatry, Indiana University School of Medicine, Indianapolis, IN, USA

**Keywords:** Dementia, Adherence, Tolerability, Pharmacogenomics

## Abstract

**Background:**

The class of acetylcholinesterase inhibitors (ChEI), including donepezil, rivastigmine, and galantamine, have similar efficacy profiles in patients with mild to moderate Alzheimer’s disease (AD). However, few studies have evaluated adherence to these agents. We sought to prospectively capture the rates and reasons for nonadherence to ChEI and determine factors influencing tolerability and adherence.

**Methods/design:**

We designed a pragmatic randomized clinical trial to evaluate the adherence to ChEIs among older adults with AD. Participants include AD patients receiving care within memory care practices in the greater Indianapolis area. Participants will be followed at 6-week intervals up to 18 weeks to measure the primary outcome of ChEI discontinuation and adherence rates and secondary outcomes of behavioral and psychological symptoms of dementia. The primary outcome will be assessed through two methods, a telephone interview of an informal caregiver and electronic medical record data captured from each healthcare system through a regional health information exchange. The secondary outcome will be measured by the Healthy Aging Brain Care Monitor and the Neuropsychiatric Inventory. In addition, the trial will conduct an exploratory evaluation of the pharmacogenomic signatures for the efficacy and the adverse effect responses to ChEIs. We hypothesized that patient-specific factors, including pharmacogenomics and pharmacokinetic characteristics, may influence the study outcomes.

**Discussion:**

This pragmatic trial will engage a diverse population from multiple memory care practices to evaluate the adherence to and tolerability of ChEIs in a real world setting. Engaging participants from multiple healthcare systems connected through a health information exchange will capture valuable clinical and non-clinical influences on the patterns of utilization and tolerability of a class of medications with a high rate of discontinuation.

**Trial Registration:**

Clinicaltrials.gov: NCT01362686

## Background

For the past 20 years, pro-cholinergic drugs such as donepezil, galantamine and rivastigmine have been considered the standard of care for mild to moderate Alzheimer’s disease (AD). These medications are not purported to alter the natural history of the disease but they are believed to offer symptomatic improvements, such as minimizing behavioral disturbances, representing one of the more difficult management problems for providers and caregivers of patients with dementia [1,2]. In a recent Cochrane Systematic Review, Birks examined the results of 13 randomized, double blind, placebo controlled trials of these agents [1]. The author concluded that these agents produced small improvements in cognitive function, activities of daily living, and behavior. Additionally, nearly one in three patients stopped treatment due to adverse effects such as nausea, vomiting, and diarrhea. The review found few studies and little evidence to suggest superiority of one of the agents over another. Other recent meta-analyses reach similar conclusions [3-5]. In 2008, we completed a meta-analysis to evaluate the efficacy of anti-dementia medications in reducing behavioral symptoms [5]. In comparison to placebo, these medications reduced behavioral symptoms with effect sizes of 0.10 to 0.16 on the Neuropsychiatric Inventory (NPI) [6] depending on the degree of dementia severity.

In the United States, the branded prescription cost for these drugs is approximately $2,500 to $3,500 per person per year [7], though the recent introduction of generic products to the market has significantly reduced the cost. However, because of limited efficacy, costs, and adverse effects, these drugs remain controversial among clinicians [2,8]. In a study of physicians’ attitudes, Franz *et al*. reported that 82% of prescribers had ambivalent or negative impressions about prescribing cholinesterase inhibitors [8]. Additionally, the United Kingdom National Health Service has debated whether to include these agents on the national formulary due to concerns about costs, safety, and effectiveness [9].

Two significant factors that likely contribute to the low rate of prescribing pro-cholinergic drugs to Alzheimer’s patients are the concern for drug-drug interactions and tolerability. Because of the frequent burden of comorbidity of the older adult population, many Alzheimer’s patients are routinely prescribed at least five drugs, and many are prescribed even more, in an attempt by providers to optimize disease state control [10]. As the number of prescribed medications increases, the frequency of daily administration requirements often increases, requiring the patient and/or caregiver to manage a more complex regimen. Many of these co-administered medications alter or compete for the activities of the hepatic drug metabolizing enzymes [11]; therefore, the pharmacokinetics of many metabolized drugs may be highly variable. This is important for the Alzheimer’s drugs donepezil and galantamine because their primary route of elimination is hepatic metabolism [12,13]. For example, paroxetine and bupropion are prescribed to Alzheimer’s patients [14] and are strong inhibitors of CYP2D6, an enzyme important for the metabolism of donepezil and galantamine [15,16]. As expected, in Alzheimer’s patients, the pharmacokinetics of these drugs shows substantial variability (for example, SD 39 to 51%) [17]. It may therefore be proposed that concurrent medications, as well as genetic variations in the drug metabolism enzymes, are a significant cause of the inter-individual variability in efficacy and tolerability of the Alzheimer’s drugs.

Comparative effectiveness research studies evaluate alternative treatments or methods in the real-world setting. Below we describe a study with a secondary aim of understanding the role of drug-drug interactions and pharmacogenomics in patients receiving therapy as routinely provided to AD patients to better understand the reasons for adherence and persistence to acetylcholinesterase inhibitors (ChEIs). We hypothesize that patient-specific clinical characteristics, including concomitant medications, comorbidity, and pharmacogenomic characteristics will predict tolerability to certain ChEIs. We will capture a broad array of phenotypic data that will be used to help optimize the therapeutic and cost effectiveness of these drugs.

## Methods/design

### Ethical approval

The study has been approved by the Institutional Review Board (IRB) of Indiana University Purdue University Indianapolis, as well as each participating healthcare system. Approval for enrollment in the study requires informed consent provided by the potential participant’s legally authorized representative.

### Study location and population

Participants are enrolled from one of four healthcare systems within the metropolitan Indianapolis area. These healthcare systems include Wishard/Eskenazi Health, Indiana University Health, St. Vincent Health, and Community Health Network systems. Each memory care practice within these healthcare systems provides services for a standardized evaluation of cognitive health, including comprehensive neuropsychologic testing, and other necessary laboratory and imaging parameters recommended for the diagnosis of cognitive impairment and dementia. A sample representation of the eligible study population, those aged 65 years and older, from all collaborating healthcare systems is provided in Table 1.

**Table 1:** Demographic description of older adult population within local healthcare systems representing the Indianapolis Discovery Network for Dementia

**Variable**	**IDND Health Care Systems**
Number aged ≥ 65 years	99,574
Number of annual outpatient visits	345,991
Mean age, yrs	74.8
African American, %	16.6%
Female, %	60.0%
ICD-9 diagnosis of dementia, %	3.4%
ICD-9 diagnosis of diabetes mellitus, %	11.6%
ICD-9 diagnosis of hypertension, %	29.6%

IDND, Indianapolis Discovery Network for Dementia.

### Study design

The design adopted in this study is a prospective, randomized open-label clinical trial intended to compare adherence to and tolerability of the three ChEIs approved for treatment of probable AD. The study will include an assessment of the primary and secondary outcome variables at baseline and weeks 6, 12, and 18. Baseline interviews will be conducted during the clinic visit in the memory care practices at the time of initiation of the ChEI, while follow up assessments will be conducted over the telephone. We hypothesize that clinical and pharmacogenomic characteristics will explain differences in tolerability among the three FDA-approved medications.

### Eligibility and enrollment

Eligible participants in this study will include those diagnosed with possible or probable AD within a memory care practice of the four healthcare systems within central Indiana. Complete eligibility criteria require (1) the provider’s intent to initiate therapy with a ChEI; (2) agreement from a caregiver to complete the study outcomes assessments; (3) access to a telephone, and (4) the ability to understand English to complete survey outcome assessments. Although the intended target population is comprised of those deemed by the physician as appropriate for initiation of a ChEI, prior exposure to this class of medications is not a contraindication for eligibility. The study will not enroll subjects with an appropriate diagnosis who are currently receiving treatment with a ChEI. In this pragmatic study, initiation of the a ChEI is at the discretion of the clinicians at each study cite. Exclusion criteria for study eligibility are simply those who have had a prior adverse drug event from one of the study medications, which resulted in the clinician’s decision to avoid another trial of a ChEI.

We are aware from our past work that passive enrollment techniques (written flyers or posters, media advertisements, provider referral) have limited effectiveness. Recognition of these enrollment barriers are an important part of the genesis of the Indianapolis Discovery Network for Dementia (IDND) [18]. Effective enrollment requires the presence of research personnel within each of the clinics and direct access to patients in collaboration with the memory care providers and leadership. To accomplish this, a study research assistant is invited into each memory care practice to obtain informed consent from each eligible patient and/or an informal caregiver for study participation. The research assistant is also responsible for conducting the telephone interviews that constitute the outcome assessments.

**Figure 1:**
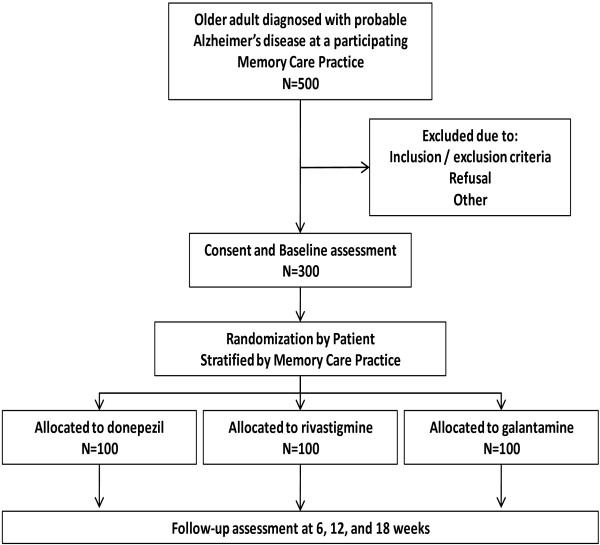
COMparative Effectiveness research Trial of Alzheimer’s disease drugs (COMET) study planned enrollment flowchart.

Among the four participating practices, there are 12 half-day clinic sessions per week. In each half-day session, providers care for approximately one to two new patients and four to five established patients. Thus, over an 18-month period, we expect approximately 1,500 potentially eligible patients, including both new and established patients. Based on IDND data, we forecast that one third of these patients (n = 500) would have a diagnosis of possible or probable AD [19]. Estimating that 40% of the targeted patients could not or would not provide permission to participate or would not meet inclusion criteria, our study anticipates enrolling the targeted 300 patients, with each practice contributing approximately 75 participants. Enrolled subjects will be followed for 18 weeks. Thus, we anticipate an enrollment rate of four to five subjects per month per memory care practice or approximately one subject per week per memory care practice (Figure 1).

### Randomization

The randomization process will allocate participants to one of three ChEIs, donepezil, rivastigmine, or galantamine, and will be stratified by location. A computer-generated randomization scheme is implemented in REDCap, the Research Electronic Data Capture web-based system for data collection available to the Clinical and Translational Science Institutions (CTSI) and is accessible online. All participating memory care practices have agreed to follow the randomization protocol for patients participating in this comparative tolerability study. Because the class of medications studied in this trial is FDA-approved, none of the allocation assignments represents experimental therapy and no patients will be allocated to placebo.

### Description of the intervention

The study intervention is limited to the initial treatment allocation, determined by the randomization method described above. Beyond the initial treatment selection, the study will use a natural treatment and management design. The memory care practice physicians will make determinations about initial drug dosage and any dosage changes and the timing of those changes. These physicians will also make the determination about whether to switch to a different ChEI, add memantine, or any other agent as needed on an individualized basis. We will, of course, monitor the frequency and content of such changes in the natural course of patient care throughout the 18-week duration of the study.

Because each study site required approval through each respective institutional review board, each study site opened enrollment at various times. Following 12 months of enrollment, six months of which included active enrollment at all study sites, a total of 80 participants have been enrolled and randomized into the study. Table 2 provides demographic data for the population completing the randomization process.

**Table 2:** Demographic description of the COMET study population

	**Overall**
	**(n = 80)**
**Gender, % (number)**	
Male	23.8 (19)
Female	76.2 (61)
**Ethnicity, % (number)**	
Hispanic	1.3 (1)
Not Hispanic	98.7 (77)
**Race, % (number)**	
African American	41.2 (33)
White	58.8 (47)
**Education, % (number)**	
Less than HS	14.1 (11)
Some HS	20.5 (16)
HS Graduate	32.1 (25)
Some college	33.3 (26)
**Marital Status, % (number)**	
Married	45.6 (36)
Widowed	39.2 (31)
Other	15.2 (12)
**Age, mean (SD)**	81.0 (8.1)
**CG relation to patient, % (number)**	
Spouse	32.9 (26)
Daughter	41.8 (33)
Son	10.1 (8)
Other	15.2 (12)
**How often CG sees patient, % (number)**	
Daily	81.0 (64)
Weekly	16.5 (13)
Several times/month	1.3 (1)
Several times/year	1.3 (1)

HS, high school; CG, caregiver.

### Evaluation/assessment parameters

#### Clinical data repository and clinical informatics tools

For more than 40 years, researchers at the Regenstrief Institute (Indianapolis, IN) have developed and operated a massive clinical informatics infrastructure, the Regenstrief Medical Record System (RMRS) [20]. Now a robust, operational health information exchange, this informatics effort created the Indiana Network for Patient Care (INPC), which serves to aggregate electronic medical record data from multiple clinical practices, laboratories, imaging centers, pharmacies, the local and state health departments and Indiana Medicaid in order to provide effective clinical care, reduce redundancies, improve healthcare quality and efficiency and reduce cost.

INPC participants deliver registration records (demographics), laboratory data, emergency department, inpatient and outpatient encounter data including free-text chief complaint, and coded diagnoses and procedures (including length of stay) for hospital admissions and emergency department visits. Some participants also deliver pathology, pharmacy, and vital signs data. Today, the INPC receives and processes clinical data from over 30 hospitals with 24 additional hospitals in process located across the state, local laboratories and imaging centers, and a few large group practices closely tied to hospital systems. The INPC also currently gathers clinical data for a number of commercial payers, including medication claims data for those receiving care within the INPC institutions as provided by Surescripts™. Table 3 summarizes data contributed to INPC by each healthcare system participating in the COMET study. A comprehensive and updated list of all contributing healthcare organizations can be found at http://www.ihie.org/Solutions/indiana-network-for-patient-care.php[21].

**Table 3:** Summary of contributed data capabilities of the Indiana Network for Patient Care (IDND)

**Data Type**	**IDND facilities participating in COMET study and providing data to INPC**			
	**Community Health System**	**Indiana University Health**	**St. Vincent Health**	**Wishard/Eskenazi Health System**
**Admission/discharge**	X	X	X	X
**Emergency Department**	X	X	X	X
**Clinic visit**	X	X	X	X
**Text reports**	X	X	X	X
**Laboratory**	X	X	X	X
**Radiology**	X	X	X	X
**Transcription**	X	X	X	X
**Cardiology**		X		X
**EKG**		X	X	
**Other**		X		X

For current and comprehensive list, visit: http://www.ihie.org/Solutions/indiana-network-for-patient-care.php. EKG, electrocardiogram.

Architecturally, the system standardizes all clinical data as it arrives at the INPC vault; laboratory test results are mapped to a set of common test codes with standard units of measure for patient care, public health and research purposes. Each institution has the same file structure in the Regenstrief system and shares the same term dictionary, which contains the codes, names (and other attributes) for tests, drugs, coded answers, et cetera. INPC allows physicians working in an emergency department and within other hospital settings in any of the participating hospitals to view a patient’s previous care information from all participating institutions as a single virtual record. INPC is a centrally managed federated clinical data repository that supports a variety of services. Currently this system is being used to provide clinical services, research data, quality reporting, public health reporting and other functions.

### Data collection

Data collection for this study will be generated from two sources. First, a research assistant will complete a telephone-based survey at baseline, 6, 12, and 18 weeks. The telephone survey will include a medication assessment and other cognitive and behavioral assessments as described below. The research assistants will be blinded to the randomization allocation and will capture study-related adverse events reported by the caregiver as well as the actions taken in response to the adverse events. All other data will come from the enhanced health information technology infrastructure of the INPC as described above.

### Primary outcome

#### Discontinuation and adherence rates

The purpose of this study is not to establish efficacy of the three medications for the indication of Alzheimer’s disease. Each of these medications already has FDA approval for AD with established efficacy parameters. The primary outcome measure is therefore the tolerability and discontinuation rate among the three medications. Tolerability will be assessed through a structured telephone interview conducted at 6, 12, and 18 weeks. Based on previous systematic reviews, the rate of discontinuation of ChEIs is reportedly in the range of 30% by 12 weeks compared with placebo [1]. We will determine the approximate date and reason for discontinuation by informal caregiver reports through the telephone-based interview at 6, 12, and 18 weeks. Any reason for discontinuation will be recorded, including adverse effects and cost.

#### Medication utilization

During active use of the study medication, rates of medication discontinuation and adherence will be assessed through electronic dispensing and claims records received from participating pharmacies and payer sources within INPC. For each patient we will complete a record of all subsequent medications used over the 18-week observation period. Prescription records for patients enrolled in the trial will be reviewed using the INPC databases to evaluate the use of psychotropic medications such as anticholinergic, antidepressant, anxiolytic, and antipsychotic agents. These psychotropic medications will be categorized based on the American Hospital Formulary Service system criteria into antidementia (including cholinesterase inhibitors or memantine), antipsychotic, antiepileptic, antidepressant, and anxiolytic agents. We will categorize the anticholinergic activities of each drug into definite or possible anticholinergic activities based on the author’s prior work [21-24].

### Secondary outcome measures

#### Neuropsychiatric inventory (NPI)

The NPI has been adopted by the Alzheimer’s Disease Cooperative Studies (ADCS) investigators to obtain information on the presence of psychopathology in behavioral areas including delusions, apathy, hallucinations, disinhibition, agitation, depression, aberrant motor behavior, anxiety, night-time behavior, and euphoria. Possible scores range from 0 to 144. The inventory is administered by the interviewer to a patient/participant’s caregiver. The NPI can be used to assess changes in the patient’s behavior over the past month or other specified time intervals. If the caregiver reports the presence of psychopathology, there are follow-up questions to assess frequency, severity, and the level of caregiver distress due to the behavior. Thus, the instrument is specifically designed to also measure caregiver distress (possible scores range from 0 to 60). The administration time is about 20 minutes. The test has excellent reliability and validity [6,25].

#### The Healthy Aging Brain Care (HABC) Monitor

Using a consortium of pilot funding from the NIA-funded IU Roybal Center, the NIMH-funded Interventions and Practice Research Infrastructure Program, and industry, a local group of investigators developed a clinical tool with a simple, user-friendly assessment to aid in the diagnosis and management of dementia symptoms. The initial content of the HABC Monitor was established during a retreat of 22 multidisciplinary experts in dementia care including representatives from IDND. The HABC Monitor is designed to be completed by the patient, a family caregiver, or a professional caregiver to provide an overall assessment of current symptoms due to dementia (as well as a measure of caregiver stress). The current HABC Monitor includes 32 items covering four relevant clinical domains of dementia symptoms: cognition, function, behavioral/psychological symptoms, and caregiver burden. Each item has four categories of responses that assess the frequency of the target problem in the past two weeks, and was designed with the capability of measuring change over time [26]. The HABC Monitor takes about five minutes to complete. The current HABC Monitor has two versions, a caregiver version and a self-report version. Notably, the instrument is specifically designed for practical application in real-world clinical practice. It is also designed to facilitate electronic capture and longitudinal tracking of these data. The HABC Monitor demonstrated good internal consistency (0.73 to 0.92), test-retest reliability, construct validity indicated by correlations with the caregiver-reported NPI total score and NPI caregiver distress score, sensitivity to three-month change compared to NPI reliable change groups, and known-groups validity indicated by significant separation of Mini-Mental Status Examination (MMSE) severity groups and clinical diagnostic groups [26]. Although not designed as a screening study, there was preliminary evidence for good operating characteristics, according to area under the receiver operator curve (AUROC) with respect to gold standard clinical diagnoses, relative to MMSE or NPI [26].

#### Process of care, health care, and mortality

The study will record sociodemographic characteristics and comorbid conditions for each patient through electronic medical records captured by the INPC. Sociodemographic status will be captured in part by capturing insurance provider. As stated previously and in Table 3, the electronic medical record provides access to diagnoses, outcomes of diagnostic testing, discharge summaries, and medications. Through the available data collection systems, this study will track multiple processes of care measures including all visits to inpatient, outpatient, and emergency department visits. In addition to the healthcare systems included in this study, the INPC allows us to track hospitalization and acute care visits from approximately 58 facilities throughout the region and state of Indiana.

### Pharmacogenomics exploratory analysis

#### Sample collection

Ethylenediaminetetraacetic acid (EDTA) blood samples are collected at two time points, prior to starting treatment, and at any follow-up appointment within the 18-week study with a minimum of one month of separation between samples. Specimens are centrifuged at room temperature within one hour of collection and plasma is stored at −80°C. DNA extraction is carried out by standard methods. For improved quality control all procedures are automatically captured and time stamped in an electronic tracking system using barcodes. All of the data are stored in a Web-based biospecimen application originally co-developed by the National Cancer Institute. This application not only tracks all procedures performed on a specimen, and the exact location and availability of specimens across individual research projects, but also links all experimental data acquired from these biospecimens.

#### Pharmacogenomic characterization

To explore the possible mechanisms that contribute to the efficacy and tolerability of donepezil, rivastigmine, and galantamine, we will conduct a pharmacogenomic analysis that focuses on the phase I hepatic metabolism of these drugs. Since the elimination of donepezil and galantamine are substantially dependent on hepatic metabolism by the cytochrome P450 2D6 and 3A4/3A5 enzymes, we will determine the activity status of each participant’s CYP2D6, CYP3A4, and CYP3A5 enzymes. The specific alleles tested will be CYP2D6: (*3, *4, *5, *6, *9 nonactive alleles), and (*10, *17, *29, and *41; reduced activity alleles); CYP3A4: *1B; CYP3A5: *3, *6, and *7 alleles [27-30]. Collectively, these alleles make up >95% of the known functional variants relevant to the hepatic metabolism of the class of acetylcholinesterase inhibitors.

Since genetic variants in the *ApoE* gene have been associated with altered susceptibility to Alzheimer’s disease, and may confer different response to the approved drugs, we will also determine the *ApoE* status for the *ApoE****ϵ****2,****ϵ****3*, and ***ϵ****4* alleles. We will determine if there is a main effect of the *ApoE* genotype on tolerability, as well as include it as a covariate in the analysis of the associations with the CYP450 genotypes. Since the hypothesis for the association between CYP450 genotypes and drug effects is through the genotype’s effects on drug pharmacokinetics, the study will also measure the drug concentrations in each participant’s plasma. Drug concentrations will be determined by the Indiana University Clinical Pharmacology Analytical Core Laboratory and will use the second sample collected. We expect that the CYP450 poor metabolizers will have higher drug concentrations, and thus more side effects and, consequently, a lower rate of adherence and persistence.

### Planned analysis

Baseline characteristics of the enrolled subjects will be compared among the three randomized groups. Any imbalances between groups will be used as covariates in subsequent analyses to reduce potential bias. All statistical analysis will be performed using a combination of SAS (SAS version 9.3, SAS Institute Inc. Cary, NC, USA) and the open-source software R (Version 2.15.2012).

### Primary analyses

To compare the primary outcome of discontinuation rates of the three study medications (donepezil, rivastigmine, galantamine) at 6, 12, and 18 weeks, we will use a generalized linear mixed model with a logit link for the binary outcome with patients as random effects. The primary predictors of interest include fixed effects of time, medication, and time-by-medication interaction, stratified by study site. Variables found to have significant mean differences between groups at baseline will be added to the basic model to see if the results remain unchanged. These analyses will be corroborated with Cox regression analyses to predict the time to discontinuation of the assigned medication as reported at the follow up interviews.

To test the difference in the outcome of medication adherence, we will compare the adherence of the randomly assigned medication for each patient using the number of doses dispensed over the 18-week follow up period divided by the number of days of follow up. Time to discontinuation will also be compared between the three randomized study groups. For count data of each medication, we will use a log-linear model to compare the amount used among the three randomized groups, using similar covariates to those for modeling medication discontinuation.

### Secondary analyses

The NPI collected at each interview and biospychosocial assessments captured by the HABC Monitor at multiple time points will be incorporated into mixed effect models to account for the repeated measurements as a function of the randomized group, time, and other covariates, with patients as random effects. The group-by-time interaction is of primary interest. The health care utilization measures for the study period will be compared among groups with nominal logistic regression with group as the primary variable of interest and other covariates as appropriate.

To examine the effects of genotypes, our initial investigation found that the donepezil metabolism pathway is CYP2D6, and the galantamine metabolism pathway is CYP3A4/5, while rivastigmine lacks a dominant metabolic pathway. CYP2D6 and CYP3A4/5 have many non-functional or reduced function alleles; similarly, many medications that may be used concomitantly by study subjects may inhibit the relevant hepatic enzymes. Study subjects will be classified as a poor metabolizer for CYP2D6 or CYP3A4/5 if he/she either has a non-functional allele or takes a strong CYP2D6 or CYP3A4/5 inhibitor, respectively. Extensive metabolizers include all others without such a variance. The binary variables of poor metabolizer/extensive metabolizer and their interactions with the assigned medications will be added to the models for the discontinuation of and adherence to the assigned medications. If the estimated between-group differences in discontinuation or adherence are reduced by the inclusion of metabolic category, then some of the patients’ variable acceptance of these medications can be attributable to their genotypes. Similarly, correlations between pharmacokinetic levels for each study drug and relevant covariates (genetics, co-medications, and other demographic social variables) will be analyzed within each drug with log-linear regression.

### Sample size and power calculations

We made an assumption that donepezil has 15% discontinuation rate by 18 weeks (conservatively low based on prior literature) [1], and rivastigmine and galantamine have 35% discontinuation rate, then the study will have 88% power to detect the difference between donepezil and each of the other two medications at two-tailed 5% significance. If rivastigmine and galantamine have discontinuation rates of 30%, then we will have 79% power to detect the difference between donepezil and these medications combined. The power for detecting significant genotype effects will be low, but the analyses as proposed will suggest whether it is worthwhile to pursue the hypothesis that genotypes and protein signaling predict the tolerability of the different study medications.

### Limitations

Although the study has several strengths, some limitations are worth noting. First, participant selection is expected to be consistent with the FDA-approved use of the study medications, which includes a diagnosis of probable AD. Such diagnoses may vary by practitioner and result in a heterogeneous mixture of participants. However, because the primary outcome and the duration of the study are focused on tolerability and not efficacy, the impact on generalizability should not be significantly compromised. Second, because electronic data is contributed from multiple institutions, certain variables may not be available for analysis. In prior work with data from INPC institutions, missing data complicates comprehensive evaluation of either study outcomes or relevant covariates. However, this particular study combines electronic data collection with human interactions through telephone interviews to supplement potentially missing data. Another limitation within the electronic database may be highlighted in the use of pharmacy dispensing or claims data as a measure of medication exposure and adherence. Although the use of dispensing and claims data allows for an objective measure of medication exposure free from recall bias, there are some limitations to this data source. Notably, such data often include only prescription medications and those included in a participating plan formulary, potentially missing medications not covered by a pharmacy benefit plan. Use of pharmacy dispensing or claims data also assumes that participants consume the medication as directed. Lastly, the use of caregiver reports to evaluate symptoms of dementia may minimize the true rate of adverse events or dementia symptoms. This may complicate the comparison of results to other studies, however caregiver reporting of adverse events will be consistently reported within this study.

## Discussion

This study contains several unique features that will contribute to our knowledge of the ChEI’s in the real world. First, we engaged the specialty care practices located within a regional health information exchange to acquire a representative mix of the population, including patients of all races and income levels in a large Midwestern city. In so doing we are able to study populations poorly represented in previous dementia studies. Additionally, by using a multi-site approach of healthcare systems involved in a health information exchange we are able to supplement data capture in the study with clinical data recorded in each electronic medical record. Third, our study objectives offer a unique opportunity to study relevant clinical and non-clinical influences on utilization of CHEI’s in a population with a high rate of discontinuation.

The design and outcomes assessed in this randomized trial will provide useful information regarding the real-world use, tolerability, and persistence with ChEI’s among older adults diagnosed with probable AD. Supplementing electronic healthcare utilization data with telephone interviews provides a wide array of relevant clinical and non-clinical variables to better understand the management of dementia in real world memory care practices. This study also has the potential to advance theories of tolerability among participants using novel covariates that may predict clinical outcomes and translate to future scientific work within this vulnerable population.

## Trial status

At the time of manuscript submission, the study has been actively enrolling participants for approximately two years, with study sites initiating enrollment procedures as IRB approval was granted. Therefore, sites have been actively enrolling subjects between 17 and 24 months. During this enrollment period, a total of 140 subjects have been enrolled, 104 have completed follow up at six weeks and 81 have completed follow up at the 18-week study endpoint. To date, completion of baseline interviews has occurred in 96% of enrolled participants and 87% of the 18-week interview, indicating a high rate of success in capturing study-related endpoints throughout the follow up period.

## Abbreviations

AD: Alzheimer’s disease; ADCS: Alzheimer’s disease cooperative studies; AUROC: area under the receiver operator curve; ChEI: acetylcholinesterase inhibitors; CTSI: Clinical and translational science institutions; EDTA: ethylenediaminetetraacetic acid; ELISA: enzyme-linked immunosorbent assay; HABC: Healthy aging braincare; IDND: Indianapolis discovery network for dementia; INPC: Indiana network for patient care; IRB: Institutional review board; MMSE: Mini-mental status examination; NPI: Neuropsychiatric inventory; REDCap: Research electronic data capture; RMRS: Regenstrief medical record system.

## Competing interests

The authors declare that they have no competing interests. Neither the funding agency nor any outside organization had a role in study design or manuscript preparation.

## Authors’ contributions

All authors have read and approved the manuscript. All authors were involved in study design and manuscript development.
